# Neutrophil-to-Lymphocyte Ratio and Absolute Lymphocyte Count as Early Diagnostic Tools for Corona Virus Disease 2019

**DOI:** 10.7759/cureus.22863

**Published:** 2022-03-05

**Authors:** Muhammad F Shahid, Asma Malik, Fuad Ahmad Siddiqi, Imran Fazal, Muhammad Hammad, Asad Saeed, Naveed Abbas

**Affiliations:** 1 Internal Medicine, Pak Emirates Military Hospital (PEMH), Rawalpindi, PAK; 2 Internal Medicine, Combined Military Hospital, Rawalpindi, PAK

**Keywords:** covid-19 nucleic acid testing, lymphocyte count, lymphopenia, neutrophils, covid-19

## Abstract

Background and objectives

In comparison to real-time polymerase chain reaction (RT-PCR) testing, blood-related parameters including absolute lymphocyte count (ALC) and neutrophil-to-lymphocyte ratio (NLR) carry an indeterminate potential in the assessment of corona virus disease 2019 (COVID-19). Our main objective was to assess their efficacy in timely identification of COVID-19 patients and to determine whether these biomarkers can be employed as an early diagnostic tool in patients presenting with symptoms suggestive of COVID-19.

Methodology

This cross-sectional study was conducted at the Emergency Department of a Tertiary Care Hospital in Rawalpindi, Pakistan from November 2020 to March 2021. Patients suspected to have COVID-19 on a clinical basis (fever, cough or shortness of breath) were selected by using convenience non-probability sampling. RT-PCR was used to diagnose COVID-19 after evaluating NLR and ALC of the sample population. An NLR = 3.5 and ALC < 1 x 10^3^ cells/mm^3^ was considered as the cut-off value. Statistical analysis was conducted via SPSS 23.0 (IBM Corp., Armonk, NY). Chi-square and independent t-tests were used to correlate various data variables, while p-value <0.05 was considered significant.

Results

Out of the 172 subjects included in the study, the mean age was 40.6 ± 10.0 years, while 51% of individuals were males. Fever was found to be the most prevalent complaint (94%). Double RT-PCR testing showed that 51.2% of the population was RT-PCR positive, having a mean ALC of 1.4 ± 0.9 x 10^3^/mm^3^, significantly lower than RT-PCR negative cases (p < 0.001). In addition, NLR was drastically elevated for RT-PCR-positive individuals (p < 0.001) while it also had a distinctly high specificity of 91.7% among COVID-19 patients. Additionally, NLR did not correlate with any of the baseline patient-related parameters (presenting complaint, age, and gender).

Conclusion

NLR and ALC are potentially efficacious measures for an early diagnosis of COVID-19, and can be possibly utilized for an early diagnosis of COVID-19 suspects.

## Introduction

Corona virus disease (COVID-19) has stringently challenged the fortitude of healthcare facilities throughout the globe, and has equivalently affected all walks of life. Ever since the pandemic began in late 2019, polymerase chain reaction (PCR) testing kits have been made available to diagnose the disease among patients suspected of clinical manifestations related to COVID-19. Ever since the emergence of COVID-19, real-time polymerase chain reaction (RT-PCR) has been widely implemented to allow a preliminary detection of COVID-19, and to help isolate RT-PCR positive individuals until a “double negative” PCR is confirmed [1]. Besides RT-PCR, several novel diagnostic approaches have been evaluated to speed up the process of identifying this viral disease as early as possible. Rapid diagnosis of COVID-19 is highly essential for those hospitalized individuals who are categorized under high-risk groups for developing serious complications [2].

At present, COVID-19 real-time PCR is considered as the first-line laboratory test for testing the clinically suspected population. In comparison to the high-resolution computed tomography (HRCT) scan, RT-PCR offers a sensitivity as low as 63% [3,4] and as high as 95% [5]. On the other hand, its specificity is estimated to be ≥95% [3,5]. In addition, several biomarkers of inflammation have been utilized to assess the status of COVID-19 among hospitalized cases [6]. Complete blood count (CBC) is perhaps one of the commonest laboratory-based tests ordered by medical practitioners. Recent literature findings have proposed that laboratory measures as straightforward as the total (TLC) and differential (DLC) leukocyte counts can be used to assess the possibility of having COVID-19. Tracking the neutrophil-leukocyte ratio (NLR) is a viable biomarker for evaluating the prognosis of different clinical conditions including many forms of cancer [7], sepsis [8], and neurodegenerative disorders [9]. Similarly, NLR has been considered as an effective tool in mapping the overall prognosis among COVID-19 patients where higher levels of this indicator lead to a drastically poor survival rate [10,11]. The efficacy of NLR has been reported to be the highest when compared with various markers of inflammation like C-reactive protein (CRP), and other CBC-related parameters [12].

To date, only a few credible studies have been conducted to ascertain whether NLR and absolute lymphocyte count (ALC) can be accurately used as an early predictor of RT-PCR positivity among COVID-19 suspects [13-15]. Furthermore, very few clinicians use this diagnostic index as an adjunct tool to RT-PCR testing. Therefore, this study has been designed to determine the effectiveness of NLR and TLC in the early detection of disease among COVID-19 suspects, and to predict whether this tool can be successfully used as a screening tool for their close contacts.

## Materials and methods

Objective

The objective of this study was to assess the effectiveness of CBC-based tools, i.e., NLR and absolute lymphocyte count (ALC) in the early detection of COVID-19 RT-PCR positive individuals.

Study design and sampling technique

This study was conducted as a cross-sectional analysis at the COVID-19 Emergency Department of a Tertiary Care Hospital in Rawalpindi (Pakistan). The study lasted for a total duration of five months from November 2020 to March 2021. The participants were selected on the basis of non-probability convenience sampling technique. The sample population comprised a total of 255 patients, all of whom had clinical manifestations suggestive of COVID-19 (persistent fever, cough and/or shortness of breath).

Inclusion and exclusion criteria

The study population was selected only if they fulfilled all of the following criteria: (1) Any of the above clinical manifestations indicative of COVID-19; (2) Duration of illness ≤ 2 days and (3) Patient age ranging from 25 to 60 years (to ensure a satisfactory infectivity rate) [16]. On the other hand, patients were excluded if they had been diagnosed with any severe co-morbidities (e.g., diabetes mellitus, hypertension, ischemic heart disease, or any other chronic disease process). Following exclusion, a total of 172 patients were included in the final statistical analysis.

Operational definitions

Clinically suspected individuals (presenting with persistent fever, cough and/or shortness of breath) were marked as “possible” cases of COVID-19.

An optimum range of NLR has been estimated to be 0.78-3.53 (Mean ± SD = 1.65 ± 1.96) [17]. In this regard, patients were defined as “potential” COVID-19 suspects if their NLR was raised above a cut-off value of 3.5. Furthermore, an ALC < 1 x 10^3^ cells/mm^3^ (normal range: 1-4 x 10^3^ cells/mm^3^) was considered as a major indicator of COVID-19.

A patient was characterized as “COVID Positive” only if they turned out a minimum of two positive Serial RT-PCR Tests.

Data collection and analysis

An informed consent was obtained from all the participants while an ethical permission was also granted from the hospital ethical committee. The demographic details, a brief history of COVID-19 related symptoms, any history of contact with COVID-19 patients or with any potential carriers of infection, and CBC parameters including the neutrophil count, ALC and NLR were entered into a pre-designed proforma. Samples for RT-PCR were obtained via nasopharyngeal swabs, and examined at the Emergency Department laboratory of the respective hospital in Rawalpindi.

The statistical parameters were analyzed by means of IBM SPSS version 23.0 (IBM Corp., Armonk, NY). Pearson’s chi-square test and independent t-test were used to assess the statistical correlation of data. Odds ratio was calculated for both ALC and NLR values. A p-value < 0.05 was considered significant. Moreover, sensitivity and specificity values were evaluated for NLR and ALC measures by using the standard formulae.

## Results

All the statistical data were obtained from a sample of 172 “possible” COVID-19 patients. Mean age was calculated to be 40.6 ± 10.0 years while approximately 51% of patients were males. More than 90% of the participants presented with fever, whereas cough was persistently present in up to 73% of the subjects. Shortness of breath was the next most common presenting complaint inflicting ~24% of individuals. Furthermore, a contact history was significant for less than 1/3rd of the study population (Table 1).

**Table 1:** Baseline Parameters of the Sample Population

Baseline Patient-related Parameters		Number of Subjects (n = 172)
Age	< 40 years	87 (50.6%)
	≥ 40 years	85 (49.4%)
Gender	Males	88 (51.2%)
	Females	84 (48.8%)
History of Contact with COVID patients	Yes	51 (29.7%)
	No	121 (70.3%)
Fever	Yes	162 (94.2%)
	No	10 (5.8%)
Cough	Yes	126 (73.3%)
	No	46 (26.7%)
Shortness of Breath	Yes	42 (24.4%)
	No	130 (75.6%)

Double RT-PCR testing confirmed COVID-19 in 51.2% of individuals of the sample population. Mean CBC values were computed for all of the participants. Mean TLC was 8.4 ± 3.4 x 10^3^/mm^3^ while mean ALC and mean neutrophil count were estimated to be 2.0 ± 1.2 x 10^3^/mm^3^ and 5.6 ± 2.6 x 10^3^/mm^3^ respectively. Mean NLR was found to be equal to 3.5 ± 2.0 which nearly falls on the predetermined cut-off value of 3.5. As depicted in Table 2, both the NLR and ALC were significantly associated with the results of COVID-19 RT-PCR (p < 0.001) where the patients having a potentially raised NLR or a low ALC had a stronger likelihood of turning out as RT-PCR positive (OR_NLR_: 16.7 and OR_ALC_: 10.1), as evaluated through unpaired t-test analysis.

**Table 2:** Blood Cell Counts and Neutrophil-to-Lymphocytic Ratio (NLR) compared with the COVID-19 RT-PCR results of the Sample Population

CBC-based Parameters	COVID PCR Result (n = 172)		Independent t-test value	p-value
	Positive (n = 88)	Negative (n = 84)		
Mean TLC (± SD) x 10^3^/mm^3^	8.0 ± 3.5	8.8 ± 3.4	-1.652	0.100
Mean Neutrophil count (± SD) x 10^3^/mm^3^	5.8 ± 2.7	5.4 ± 2.5	1.039	0.300
Mean ALC (± SD) x 10^3^/mm^3^	1.4 ± 0.9	2.6 ± 1.2	-7.743	< 0.001
Mean NLR (± SD)	4.8 ± 2.0	2.3 ± 1.0	10.597	< 0.001

The mean NLR did not show any substantial variation with respect to the baseline features of the patients including their age, gender, and presenting clinical manifestations (Table 3). Although the patients having fever, cough or shortness of breath had a relatively higher NLR, these results were not statistically significant.

**Table 3:** Determinants of the Neutrophil-to-Lymphocyte Ratio (NLR)

Mean Neutrophil-to-Lymphocyte Ratio (NLR ± SD)	Patient-related characteristics		Independent t-test value	p-value
	Age		-0.196	0.845
	< 40 years: 3.5 ± 1.8	≥ 40 years: 3.6 ± 2.2		
	Gender		0.640	0.523
	Males: 3.6 ± 2.1	Females: 3.4 ± 1.9		
	Fever		0.444	0.658
	Yes: 3.6 ± 2.0	No: 3.3 ± 2.3		
	Cough		0.846	0.399
	Yes: 3.6 ± 2.0	No: 3.3 ± 2.0		
	Shortness of Breath		1.731	0.085
	Yes: 4.0 ± 2.1	No: 3.4 ± 1.9		

In terms of establishing an early diagnosis of COVID-19, NLR had a remarkably high specificity of 91.7% whereas this CBC measure had only a moderately high sensitivity (60.2%). For ALC, specificity was 96.4% whereas sensitivity stood quite low at 27.3%.

## Discussion

This study has quantitatively measured the efficacy of hematological testing in predicting the probability of positive COVID-19 RT-PCR results. The authors have statistically elaborated that both ALC and NLR are fairly valuable tools in the early detection of corona virus, and that none of the baseline parameters are correlated with the variation in NLR. Having a remarkably high specificity, both ALC and NLR are useful measures for ruling out a diagnosis of COVID-19 among the clinical suspects of the infection. These results hold significance for directing the referral of these potential COVID-19 cases for an immediate RT-PCR test. Furthermore, NLR and ALC hold substantial relevance as comparatively cheaper as well as readily available diagnostic tools for COVID-19.

Although there has not been much progress in the evaluation of NLR or ALC as a screening tool for COVID-19, still a number of authors have independently declared these two parameters as viable instruments for predicting the overall prognosis of patients. In contrast to moderately ill COVID-19 patients, Liu et al. have found that severely ill cases possess a mean NLR which is approximately four times higher than the former [18]. Moreover, severely raised NLR had a remarkable association with multiple markers of inflammation including D-dimers, CRP, and fibrinogen. In the same study, the authors also concluded that NLR > 5.0 was a major determinant of critical COVID-19, thereby requiring a broad-scale emergency intervention. Similar findings were obtained from another study where NLR elevated above 7.4 was targeted as a major predictor for COVID-19-related mortality [19]. In another study conducted by Yan et al. [20], it was revealed that a high NLR (>11.75) was significantly linked to a potentially greater risk of mortality. A comparable effectiveness of NLR as a prognostic marker for COVID-19-related mortality was also reported by Jimeno et al. [21], while another study comprising 169 COVID-19 patients has declared its own cut-off value for NLR to be around 3.9 [22].

Viral infections, in general, have often been proposed to induce secondary lymphopenia among the host organisms. Lymphopenia has been attributed to the proinflammatory state of body during the course of COVID-19 infection. A systemic cytokine storm is characterized by markedly raised levels of interleukins (IL-2, IL-6, and IL-7), and tumor necrosis factor-alpha (TNF-α), which contribute to the pathogenesis of lymphocyte apoptosis. Furthermore, it has been hypothesized that the virus exerts its cytotoxicity directly on the lymphocytes, which is mediated through angiotensin-converting enzyme-2 (ACE-2) receptors expressed on cellular membranes of the latter [23]. In addition, COVID-19 has also been associated with a reduction in the overall rate of lymphopoiesis within the lymphoid organs, i.e., lymph nodes, bone marrow and thymus. This has been mainly postulated to be linked to its direct suppression of the hematopoietic stem cells [24]. Another plausible mechanism of COVID-19-induced lymphopenia is a series of metabolic derangements including increased serum lactate levels [25].

Low absolute lymphocytic counts have been implicated in COVID-19 by several authors. In one study, lymphopenia was a consistent laboratory finding encountered within the blood of up to 82% of deceased COVID-19 cases [26]. A retrospective analysis carried out by Güneysu et al. found that the patients having a lymphocyte count < 1.12 x 10^3^/mm^3^ at the time of their emergency admission were predominantly at high risk of facing an early mortality [22]. Contrariwise, a gradually rising ALC is believed to be a vital indicator of recovery from severe COVID-19 [27].

Only a handful of authors have hypothesized the applicability of ALC as an early diagnostic marker for COVID-19. One meta-analysis showed that ALC was the single most efficient laboratory assessment tool in emergency scenarios where more than 50% of patients had a subnormal lymphocyte count [28]. In another review, the authors determined that ALC evaluation had a sensitivity as high as 64% for ascertaining the diagnosis of COVID-19 [29]. A few authors have also accommodated this parameter into their diagnostic algorithms for corona virus disease [30]. Nonetheless, large-scale multi-center trials are warranted to declare ALC or NLR as a prominent diagnostic parameter for detecting COVID-19 in the emergency department.

Limitations

Although the sample size was statistically calculated for this study, one of its major drawbacks is the relatively limited sample population which was confined to only a single medical facility. This could have potentially influenced the statistical significance of our results. Additionally, different RT-PCR kits show remarkable differences in terms of their relative sensitivity and specificity, and these observations are quite well-documented [31, 32]. This could have impacted the validity of our statistical conclusion. Moreover, it is acknowledged that the results could have been further strengthened by an additional monitoring of the inflammatory biomarkers (e.g., D-dimers, etc.); however, the authors did not resort to the latter.

## Conclusions

An increased neutrophil-to-lymphocyte ratio, as well as lymphopenia, are substantially effective tools in the early detection of COVID-19. With larger trials, it might become possible to consistently use these laboratory measures prior to referring patients for COVID-19 RT-PCR testing. Furthermore, a significantly high specificity for NLR has the potential to allow its application for ruling out the probability of a positive RT-PCR result. Our study can significantly impact the care of COVID patients in resource-poor settings where it can be utilized to triage patients most in need of a PCR test for the diagnosis of COVID-19 infection.
